# Notes in Reply to Mr. Hall's Strictures on the Criticisms of F.R.I.B.A. "Nos. 1 and 2."

**Published:** 1912-04-27

**Authors:** 


### NOTES IN REPLY TO MR. HALL'S STRICTURES ON THE CRITICISMS OF F.R.I.B.A. "NOS. 1 AND 2."

F.R.I.B.A. "No. 1" writes under date April 22:—

"In reply to Mr. E. T. Hall's letter of the 15th inst., in which he takes exception to certain parts of my report, I have to say that the opinions expressed therein are based upon a careful examination of the documents in question, and I have nothing to withdraw or modify, with this single exception: that as to the cost; although the sum of £35,000 is not definitely stated as a maximum, still it would undoubtedly be and probably was taken so by most of the competitors. The fact that preference is to be given to a design within that sum if possible clearly imposes a very definite limit upon the competitors, and it seems to me to be straining language to say that there is not one word in the conditions about fixing £35,000 as the total or maximum cost."

F.R.I.B.A. "No. 2" sends the following notes on Mr. Hall's letter, with the conditions and a plan of the site:—

"Cost.—The sum of £35,000 was mentioned as the amount in the committee's mind as the maximum. Further, it was stated, as quoted by Mr. Hall, 'Preference will be given to a design within that sum, if possible, but competitors must use their discretion in designing if they think that sum insufficient.' What other inference could be drawn from these words other than that the sum was definite, especially when it is further stated that 'Preference will be given to a design within that sum, if possible.' Mr. Hall's quotation of the condition referring to cost is correctly stated, except that a comma is introduced after 'That sum' which does not occur in the original. Readers can judge what the words imply; otherwise, why should any sum be stated at all? This is fair comment.

"Site.—In reference to the statement as to levels, it will be remembered that they formed a most complex problem. Note 2, it will be seen, refers to the printed *Conditions* and not to the Answers to Competitors on which Mr. Hall relies. The plan of the site had, of course, the levels marked thereon, but from the entire absence of reference in the conditions to the extreme variation of surface they might be read as if the site were level; for instance, various requirements were made which practically suggested that there were no falling levels to consider; certainly full consideration of requirements as to levels were not stated. The number of questions asked and subsequent replies, varying the conditions, show how the level difficulty must have agitated the struggling competitors through want of distinct statement of the difficult nature of the site and of the stipulated requirements of space and cost. No comment was made by 'No. 2' upon the planning, but Mr. Henman has furnished twenty-five questions in reference to this, but makes only slight reference to the important problem of levels."

[With regard to Mr. E. T. Hall's demand for a withdrawal of, and an apology for, statements contained in our issue of March 2, 1912, we publish with pleasure his letter of April 15, 1912, *in extenso*, and the observations of our contributors, the writers of the special report No. 1 and the note No. 2 on that letter. We do not find in our own preface to the special report and note any statement which we ought to withdraw or for which we can offer an apology to Mr. Hall. Of the points raised by Mr. Hall so far as they are not dealt with by our correspondents "Nos. 1 and 2," we may point out, taking them under the numbers given in his letter, as to

(1) That our article of March 2 as circulated referred to "a lamentable lack of knowledge on the part of their advisers," not *adviser*, which would be a printer's error if his copy contains it, and so did not "only refer to me," *i.e.*, to Mr. Hall, as he believes.

(2) The principle laid down by us in our editorial preface as to a committee's instructions binding the assessor is the only right one, but in view of the actual wording of Condition 12 it would appear that Mr. Hall was not specifically instructed to reject plans if the sum of £35,000 was exceeded by any competitor in his estimates of cost. Hence our contention would not directly apply to the Hastings Competition.—Ed., The Hospital.]

